# Oral epithelial dysplasia detection and grading in oral leukoplakia using deep learning

**DOI:** 10.1186/s12903-024-04191-z

**Published:** 2024-04-09

**Authors:** Jiakuan Peng, Ziang Xu, Hongxia Dan, Jing Li, Jiongke Wang, Xiaobo Luo, Hao Xu, Xin Zeng, Qianming Chen

**Affiliations:** 1 State Key Laboratory of Oral Diseases, National Clinical Research Center for Oral Diseases, Research Unit of Oral Carcinogenesis and Management, Chinese Academy of Medical Sciences, West China Hospital of Stomatology, Sichuan University, Chengdu Sichuan, China; 2 Key Laboratory of Oral Biomedical Research of Zhejiang Province, Affiliated Stomatology Hospital, Zhejiang University School of Stomatology, Hangzhou, Zhejiang China; 3 Department of Stomatology, North Sichuan Medical College, Nanchong, Sichuan, China

**Keywords:** Oral leukoplakia, Oral epithelial dysplasia, Whole-slide image, Tissue microarray, Computational pathology, Deep learning

## Abstract

**Background:**

The grading of oral epithelial dysplasia is often time-consuming for oral pathologists and the results are poorly reproducible between observers. In this study, we aimed to establish an objective, accurate and useful detection and grading system for oral epithelial dysplasia in the whole-slides of oral leukoplakia.

**Methods:**

Four convolutional neural networks were compared using the image patches from 56 whole-slide of oral leukoplakia labeled by pathologists as the gold standard. Sequentially, feature detection models were trained, validated and tested with 1,000 image patches using the optimal network. Lastly, a comprehensive system named E-MOD-plus was established by combining feature detection models and a multiclass logistic model.

**Results:**

EfficientNet-B0 was selected as the optimal network to build feature detection models. In the internal dataset of whole-slide images, the prediction accuracy of E-MOD-plus was 81.3% (95% confidence interval: 71.4–90.5%) and the area under the receiver operating characteristic curve was 0.793 (95% confidence interval: 0.650 to 0.925); in the external dataset of 229 tissue microarray images, the prediction accuracy was 86.5% (95% confidence interval: 82.4–90.0%) and the area under the receiver operating characteristic curve was 0.669 (95% confidence interval: 0.496 to 0.843).

**Conclusions:**

E-MOD-plus was objective and accurate in the detection of pathological features as well as the grading of oral epithelial dysplasia, and had potential to assist pathologists in clinical practice.

**Supplementary Information:**

The online version contains supplementary material available at 10.1186/s12903-024-04191-z.

## Background

Oral leukoplakia (OLK) is defined as white plaques or patches that cannot be wiped out of oral mucosa having excluded other known diseases by histopathology examination [1]. OLK is one of the most common oral potentially malignant disorders (OPMDs) and OLK has an appropriate malignant transformation rate of 1.1%∼40.8% [2]. One of the most important indicators of the malignant transformation of OLK is oral epithelial dysplasia (OED) [3]. OED is a pathology term which is defined as abnormal architectural and cytological changes within mucosal epithelium of the oral cavity [4]. The presence of OED in OLK correlates with an increased risk of progression to carcinoma, and the higher degree of OED, the higher risk of progression [4]. Most of the time, the severity of OED is divided into three levels (mild, moderate and severe dysplasia) according to the World Health Organization (WHO) criteria (2022 version) [5]. Although there is a binary grading system mentioned by the criteria, the validation of this system against malignant transformation remains outstanding, limiting its application [6, 7]. The 3-tiered grading criteria focus on the range or affected layers of certain pathological features, including architectural and cytological features [5]. There were significant changes of pathological features from the 2017 to the 2022 version [5, 8, 9]. Notably, the 2017 version included 16 features, whereas the 2022 version had 11 additional features [5, 8]. The added features were “altered keratin pattern for oral sub-site”, “verrucous or papillary architecture”, “extension of changes along minor gland ducts”, “sharply defined margin to changes”, “multiple different patterns of dysplasia”, “multifocal or skip lesions”, “expanded proliferative compartment”, “basal cell clustering/nesting”, “single cell keratinization”, “apoptotic mitoses”, “increased nuclear size” [5]. If there is no presence of OED in OLK, the diagnosis of OLK is often given as hyperplasia or nondysplasia [10]. Currently, the detection and grading of OED severity is poorly reproducible between observers. One more obstacle that makes OED grading difficult is that visual inspection of tissue slides is a repetitive and time-consuming task for pathologists [11]. With the need of precision medicine increasing, early detection and grading of OED in OLK patients has become critical [12]. A more objective OED detection and grading approach could be beneficial to oral pathologists and OLK patients.

Deep learning algorithms, especially convolutional neural networks (CNNs), can extract basic features of images that contain rich information, for example, from whole-slide images (WSIs). Therefore, CNNs have been used in a wide range of medical image analysis tasks, especially in cancer diagnosis [11, 13–15], molecular subtype classification [11, 16–19] and survival prediction [16, 20–23]. In recent years, several studies have explored the effect of deep learning algorithms on the prediction of OED from OPMD whole-slide images. One of the studies focused their work on predicting the severity of OED using only one CNN model and the accuracy of their CNN model reached 89.3% in an internal test dataset, at the patch-level [24]. A similar work compared two CNNs, DeepLabv3 + and UNet++, revealing that DeepLabv3 + achieved an accuracy of 93.3% [25]. In a related study, seven CNNs were utilized on OED datasets annotated with binary grading system, resulting in EfficientB0 having comparable metrics and the lowest loss among all CNNs [26]. However, the pathological features of OED, which are of great importance to OED grading, were ignored in previous studies. In a recent work, peri-epithelial lymphocytes were used to predict the malignant transformation of OED [27], but those features were still not taken into account. Furthermore, there is no convenient application that could assist pathologists in clinical practice.

In this study, we aimed to apply computational pathology methods including deep learning algorithms to OED detection and grading in OLK whole-slide images, in order to establish an objective, accurate and useful computational detection and grading system for OED.

## Methods

### Data acquisition

This study was approved by the Ethics Committee of West China Hospital of Stomatology, Sichuan University (WCHSIRB-D-2022-006). In this observational study, we collected diagnostic slides of OLK patients from oral medicine department of West China Hospital of Stomatology between 2013 and 2018. The patients were included based on the following criteria: (a) age > 18 years; (b) suspicion of OLK through clinical examination; and (c) confirmation of OLK through histopathological examination. There were 56 WSIs of 56 OLK patients that were included in this study. Additionally, we collected four OLK tissue microarrays (TMAs) from Chinese Academy of Medical Sciences Research Unit of Oral Carcinogenesis and Management. The TMAs included 229 samples of 93 OLK patients between 2004 and 2014, since some patients had multiple OLK lesions and all available tissue samples from different lesions of the same patient were included in this study. Slide preparations were completed in oral pathology department of West China Hospital of Stomatology and Chinese Academy of Medical Sciences Research Unit of Oral Carcinogenesis and Management, respectively. All slides were crafted from formalin-fixed paraffin-embedded tissue, and were hematoxylin & eosin stained. Whole-slide images were scanned using Leica APERIO VERSA 8 FL scanner and Aperio ImageScope software.

**Fig. 1:**
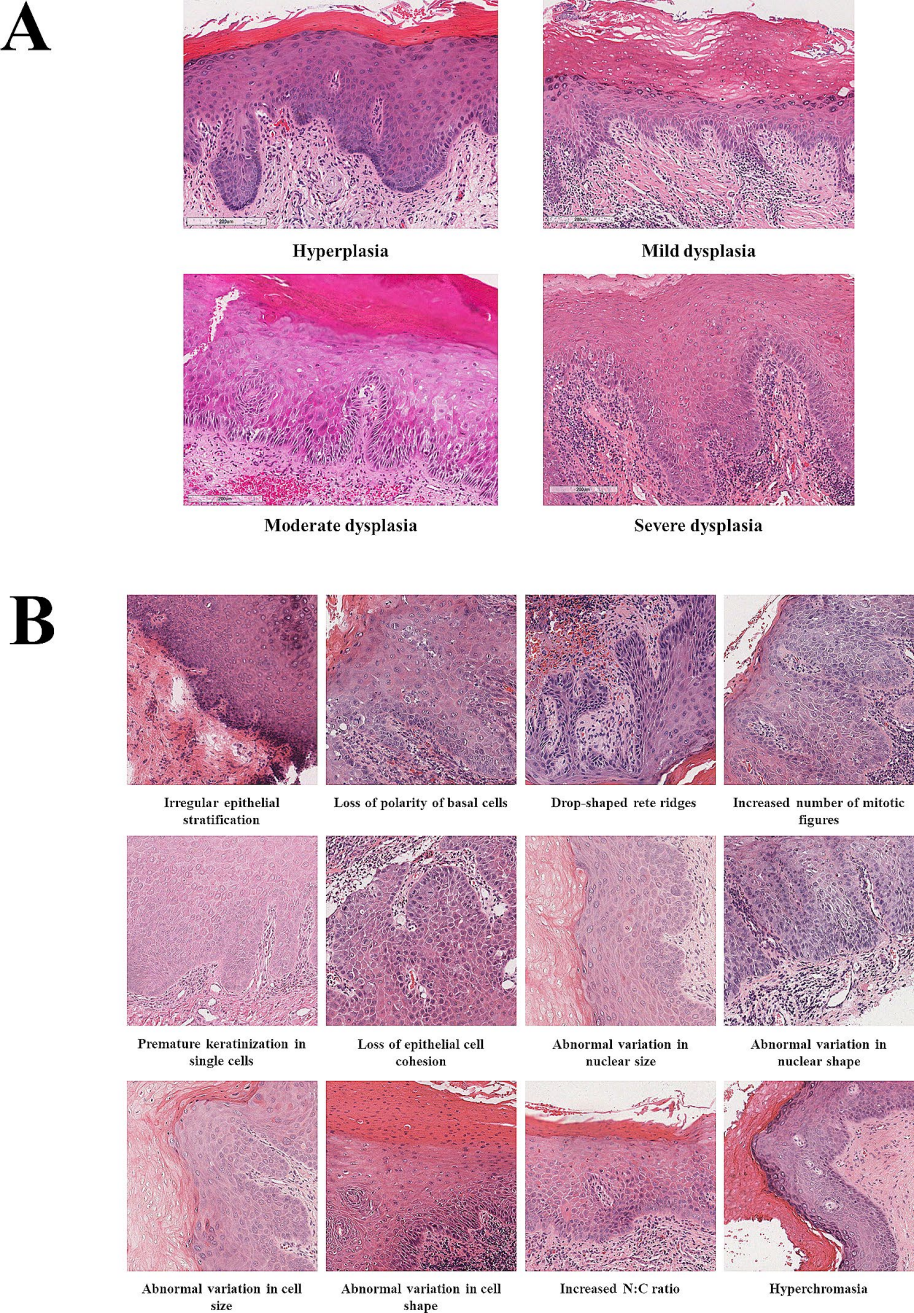
Example images of this study. (**A**) hyperplasia, mild dysplasia, moderate dysplasia, severe dysplasia in oral leukoplakia; (**B**) 12 pathological features of oral epithelial dysplasia which were included in this study

Dysplasia annotations were performed on the 56 WSIs and 229 tissue microarray images and were completed by two oral pathologists with 20-year experience who were blind to any clinical information of the patients, following the WHO criteria (2017 version) [8]: mild dysplasia can be defined by cytological changes limited to the basal third, moderate dysplasia by extension into the middle third, severe dysplasia by extension into the upper third, and the presence of architectural changes may increase the level. Any disagreement between the two observers was solved by an expert oral pathologist with 30-year experience. There were four labels given to 56 whole-slide images: hyperplasia, mild dysplasia, moderate dysplasia and severe dysplasia. A typical example of these labels is shown in Fig. 1A.

Annotations with “yes” or “no” to 16 OED pathological features were also completed by two oral pathologists with 20-year experience who were blind to any clinical information of the patients, following WHO criteria (2017 version). Any disagreement between two observers was solved by an expert oral pathologist with 30-year experience. There were 1,000 image patches (images originated from whole-slides) with annotation for each pathological feature. Pathological features which had a positive frequency less than 5 were excluded. Four pathological features were removed: abnormally superficial mitotic figures, keratin pearls within rete ridges, atypical mitotic figures, increased number and size of nucleoli. A typical example of these pathological features is shown in Fig. 1B.

### Image preprocessing

We applied non-overlapping image tiling to the slides and microarrays to split images into patches with Python module Openslide 1.1.1. Patches that contained over 50% background pixels (brightness > 220/255) were excluded [11]. To test the effect of differently scaled image inputs, we attempted to train networks using two different resolutions (224 pixels & 512 pixels) [16, 21] and two different magnifications. (10× & 20×) [16, 28] Through this process a total number of approximately 208,000 image patches were generated (approximately 161,000 WSI patches and approximately 47,000 TMA patches) (details are shown in Supplementary Table 1). The patches were labeled using the same labels as the parent slides. The size of 1000 images with pathological feature annotation was 224 pixels.

### Model training and validation

Two sorts of deep learning models were trained and validated: OED grading models and OED feature detection models.

For OED grading models, we evaluated four CNNs: ResNet-50 [29], Inception-V4 [30], ShuffleNet-V2 [31] and EfficientNet-B0 [32], to select the best network for OED grading. Grid search strategy was applied to the model selection procedure [33]. All networks were trained from scratch using randomized initial weights, with a mini-batch size of 80. The maximum number of epochs was set to 100 for every model. A model was selected only if it reached the lowest cross entropy loss in the validation dataset. The RMSprop (root mean squared propagation) optimizer was used as the optimizer of stochastic gradient descent algorithm and the learning rate was set to 1 × 10^− 4^ [34]. The patches were divided into a train set of 60% patches, a validation set of 15% and a test set of 25%.

Following CNN selection, we used the best CNN to train OED feature detection models from scratch. As the number of images with pathological feature annotation was relatively small, the maximum number of epochs was set to 200, and the learning rate of RMSprop was set to 1 × 10^− 6^, to prevent gradient explosion [35]. The other parameters remained unchanged during the training of OED feature detection models. Image distribution was in concordance with OED grading models, as 60% for train, 15% for validation and 25% for test.

Model training and validation were implemented on a workstation with four NVIDIA Tesla K80 graphics processing units, using Python 3.6.8 with modules Tensorflow 2.1.0, Openslide 1.1.1, Scikit-learn 0.23.1 and relative dependencies.

### Establishment of the comprehensive detection and grading system

The fully connected layers of OED feature detection models were removed, and the activation scores (range: 0 ∼ 1) were extracted from them. These scores represent the probability of positive predictions of the pathological features at the patch-level. To better interpret the scores, we amplified them 100 times (range: 0 ∼ 100). Slide-level scores were calculated using an average of all the patch-level scores included in a single whole-slide image. Slide-level scores were used as the variables of a multiclass logistic model (ordinal logistic model) for OED grading:

1 $$\text{l}\text{o}\text{g}\text{i}\text{t}\left(\sum _{i=1}^{n}{p}_{i}\right)={\alpha }_{n}+\sum _{j=1}^{12}{\beta }_{j}\times {Score}_{j}$$

**Fig. 2:**
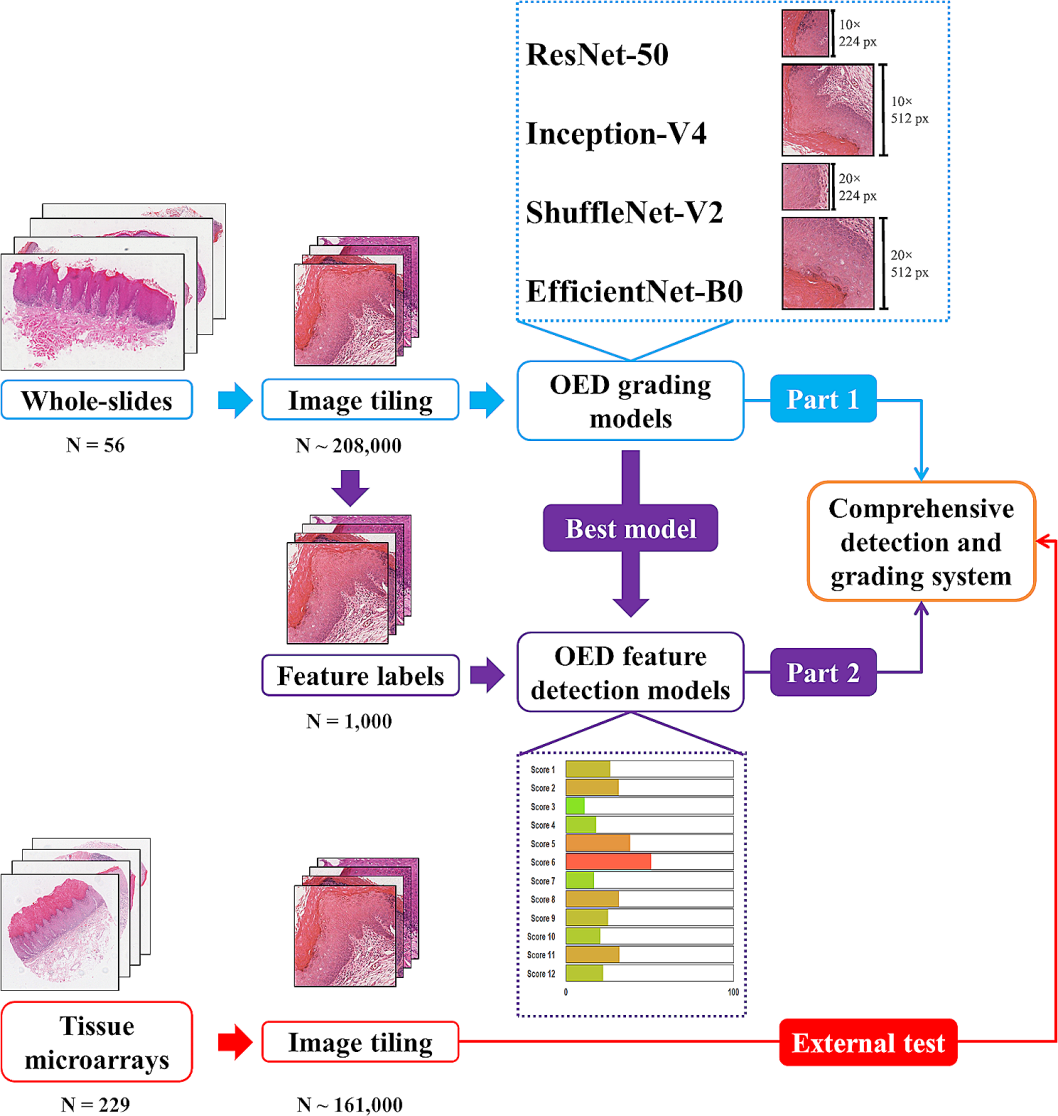
The development of the OED grading system. OED: oral epithelial dysplasia

The performance of this model was evaluated in 56 OLK whole-slides as well as 229 TMA images, at the slide level. Additionally, the performance of this model was compared with 3 junior oral pathologists who have less than 5 years’ experience. The junior pathologists were assigned to conduct OED grading following the WHO criteria (2017 version) as well. The establishment of this comprehensive detection and grading system can be summarized in Fig. 2.

### Development of a detection and grading application

We used PyQt5 to develop an application for OED grading and OED feature detection. To integrate the prediction results, heatmaps were generated using the values of categorical softmax function. Then we assembled all foreground patches (background pixels < 50%) according to the original spatial coordinates.

### Statistical analysis

The baseline information of OLK patients was tested using Kruskal-Wallis test (continuous variables) or Fisher’s test (categorical variables), two-tailed *P* value less than 0.05 was considered significant. The diagnostic accuracy indicators including accuracy, sensitivity, specificity and area under the receiver operating characteristic curve (AUC) in test datasets were used to assess the performances of trained networks. The confident intervals (CIs) of these metrics were calculated using the bootstrapping method. As the labels had four categories, these metrics were calculated as averages. The accuracy and AUC were the primary criteria for evaluation. All the statistical analysis was performed with Python 3.6.8 and R 3.6.1.

## Results

### The baseline of OLK patients

Among OLK patients whose slide images were used for training, there were 22 male patients and 34 female patients; an average age of the patients was 56.9 (± 11.8) years; and the most frequently affected anatomic site was tongue, followed by buccal mucosa, gingiva and floor of mouth. Overall, the baseline information was in consistence with the general clinical epidemiological profile of OLK, and the distribution was not significantly biased (Table 1). Among TMA cases, there were 42 male cases and 51 female cases; an average age of the cases was 58.5 (± 11.8) years; and the most frequently affected anatomic site was buccal mucosa, followed by tongue, palate, gingiva and floor of mouth. The affected sites were absent in 36 (15.7%) of 229 TMA images (Table 2).

**Table 1:** Baseline information of OLK patients

	Hyperplasia	Mild dysplasia	Moderate dysplasia	Severe dysplasia	Total	*P*
Number of slides	16	21	12	7	56	
Sex						**0.48**
Male	8 (50.0%)	8 (38.1%)	5 (41.7%)	1 (14.3%)	22 (39.3%)	
Female	8 (50.0%)	13 (61.9%)	7 (58.3%)	6 (85.7%)	34 (60.7%)	
Age	53.7 ± 13.4	57.7 ± 10.0	57.2 ± 13.8	61.7 ± 9.8	56.9 ± 11.8	**0.56**
Site						**0.65**
Buccal mucosa	4 (25.0%)	8 (38.1%)	4 (33.3%)	4 (57.1%)	20 (35.7%)	
Tongue	8 (50.0%)	11 (52.4%)	8 (66.7%)	3 (42.9%)	30 (53.6%)	
Gingiva	3 (18.8%)	2 (9.5%)	0 (0.0%)	0 (0.0%)	5 (8.9%)	
Floor of mouth	1 (6.2%)	0 (0.0%)	0 (0.0%)	0 (0.0%)	1 (1.8%)	

Categorical variables were demonstrated as frequencies and percentages, and were tested by Fisher’s test; continuous variables were demonstrated as averages and standard deviations, and were tested by Kruskal-Wallis test. OLK: oral leukoplakia

**Table 2:** Baseline information of TMA cases

	Hyperplasia	Mild dysplasia	Moderate dysplasia	Severe dysplasia	Total	*P*
Number of slides	124	43	51	11	229	
Sex						**0.55**
Male	61 (49.2%)	18 (41.9%)	20 (39.2%)	6 (54.5%)	105 (45.9%)	
Female	63 (50.8%)	25 (58.1%)	31 (60.8%)	5 (45.5%)	124 (54.1%)	
Age	57.8 ± 12.2	59.0 ± 13.0	60.8 ± 10.0	58.8 ± 9.6	58.7 ± 11.8	**0.46**
Site						***< 0.001***
Buccal mucosa	57 (46.0%)	29 (67.4%)	18 (35.3%)	7 (63.4%)	111 (48.5%)	
Tongue	33 (26.6%)	9 (20.9%)	12 (23.5%)	1 (9.1%)	55 (24.0%)	
Gingiva	9 (7.3%)	2 (4.7%)	0 (0.0%)	0 (0.0%)	11 (4.8%)	
Floor of mouth	0 (0.0%)	0 (0.0%)	0 (0.0%)	2 (18.2%)	2 (0.9%)	
Palate	14 (11.3%)	0 (0.0%)	0 (0.0%)	0 (0.0%)	14 (6.1%)	
*Missing*	11 (8.9%)	3 (7.0%)	21 (41.2%)	1 (9.1%)	36 (15.7%)	

Categorical variables were demonstrated as frequencies and percentages, and were tested by Fisher’s test; continuous variables were demonstrated as averages and standard deviations, and were tested by Kruskal-Wallis test. TMA: tissue microarray

### Selection of CNNs

In the test datasets, four CNNs performed well when trained with 20×, 224-pixel patches. EfficientNet-B0 trained with 20×, 224-pixel patches was the best model in comparison with others (average accuracy = 97.5%, Table 3). Furthermore, we calculated the AUCs of 16 models, and found that EfficientNet-B0 trained with 20×, 224-pixel patches outperformed others as well (average AUC = 0.993, Table 3). The best parameters of EfficientNet-B0 and Inception-V4 were 20×, 224 pixels, and the best parameters of ResNet-50 and ShuffleNet-V2 were 10×, 224 pixels.

**Table 3:** The performances of convolutional neural networks

	Accuracy (%)	95% CI (%)	AUC	95% CI
ShuffleNet-V2				
224 px & 10×	91.1	(90.5, 91.8)	0.957	(0.952, 0.962)
224 px & 20×	89.0	(88.7, 89.4)	0.957	(0.955, 0.959)
512 px & 10×	72.6	(70.3, 75.0)	0.729	(0.692, 0.763)
512 px & 20×	80.8	(79.8, 81.8)	0.809	(0.794, 0.823)
ResNet-50				
224 px & 10×	90.6	(89.9, 91.3)	0.954	(0.949, 0.960)
224 px & 20×	88.0	(87.6, 88.4)	0.930	(0.926, 0.933)
512 px & 10×	72.6	(70.3, 75.0)	0.633	(0.598, 0.666)
512 px & 20×	85.1	(84.2, 86.0)	0.894	(0.884, 0.903)
Inception-V4				
224 px & 10×	93.8	(93.3, 94.4)	0.979	(0.976, 0.983)
224 px & 20×	94.9	(94.6, 95.1)	0.987	(0.986, 0.988)
512 px & 10×	75.2	(73.0, 77.5)	0.603	(0.565, 0.640)
512 px & 20×	82.5	(81.5, 83.5)	0.772	(0.755, 0.788)
EfficientNet-B0				
224 px & 10×	92.8	(92.3, 93.4)	0.966	(0.961, 0.971)
224 px & 20×	97.5	(97.3, 97.6)	0.993	(0.992, 0.994)
512 px & 10×	76.6	(74.4, 78.9)	0.741	(0.709, 0.774)
512 px & 20×	89.6	(88.8, 90.4)	0.928	(0.919, 0.937)

CI: confidence interval; AUC: area under the receiver operating characteristic curve; px: pixel

Therefore, we selected EfficientNet-B0 as the best CNN for OED grading, and referred to it as **E-MOD** (**E**fficientNet-B0 **M**odel for **O**ED **D**iagnosis). E-MOD had an average accuracy of 97.5% (95% CI: 97.3%∼97.6%), an average AUC of 0.993 (95% CI: 0.992 ∼ 0.994), an average sensitivity of 94.7% (95% CI: 94.2%∼95.3%), and an average specificity of 98.3% (95% CI: 98.1%∼98.4%).

We used TMA images to test the robustness of E-MOD. At the patch-level, E-MOD had an average accuracy of 90.9% (95% CI: 90.2%∼91.6%) and an average AUC of 0.875 (95% CI: 0.865 ∼ 0.885). The predictions at the slide-level were calculated according to the maximum proportion of each category. At the slide-level, E-MOD had an average accuracy of 63.5% (95% CI: 57.4%∼69.6%), an average AUC of 0.673 (95% CI: 0.484 ∼ 0.824), an average sensitivity of 86.1% (95% CI: 81.2%∼90.6%), and an average specificity of 64.0% (95% CI: 56.5%∼71.0%).

### Testing pathological feature detection models

The performances of 12 pathological feature detection models are shown in Table 4. The median accuracy of the models was 79.7% and the median AUC was 0.766, suggesting that EfficientNet-B0 was accurate in the detection of OED features. After training 12 pathological feature detection models with EfficientNet-B0, the softmax activation scores were extracted from the models. Therefore, we obtained 12 scores as the variables of a multiclass logistic model.

**Table 4:** The performances of 12 pathological feature detection models

Pathological Feature	Accuracy (%) (95% CI)	AUC (95% CI)
**Irregular epithelial stratification**	78.2(67.3 ∼ 89.1)	0.768 (0.576 ∼ 0.930)
**Loss of polarity of basal cells**	79.3 (62.1 ∼ 93.1)	0.722 (0.578 ∼ 0.813)
**Drop-shaped rete ridges**	86.9 (78.7 ∼ 95.1)	0.900 (0.814 ∼ 0.967)
**Increased number of mitotic figures**	81.7 (71.7 ∼ 91.7)	0.806 (0.671 ∼ 0.927)
**Premature keratinization in single cells**	96.9 (92.2 ∼ 98.4)	0.734 (0.544 ∼ 0.856)
**Loss of epithelial cell cohesion**	98.4 (93.4 ∼ 98.4)	0.983 (0.948 ∼ 1.000)
**Abnormal variation in nuclear size**	67.6 (51.4 ∼ 81.1)	0.725 (0.612 ∼ 0.818)
**Abnormal variation in nuclear shape**	78.9 (65.8 ∼ 92.1)	0.776 (0.600 ∼ 0.923)
**Abnormal variation in cell size**	80.0 (68.9 ∼ 91.1)	0.883 (0.737 ∼ 0.993)
**Abnormal variation in cell shape**	88.6 (79.5 ∼ 97.7)	0.764 (0.646 ∼ 0.876)
**Increased N: C ratio**	62.5 (51.5 ∼ 79.5)	0.742 (0.604 ∼ 0.796)
**Hyperchromasia**	65.4 (51.9 ∼ 78.8)	0.679 (0.587 ∼ 0.759)

AUC: area under the curve; CI: confidence interval

### Establishment of a comprehensive grading system

**Fig. 3:**
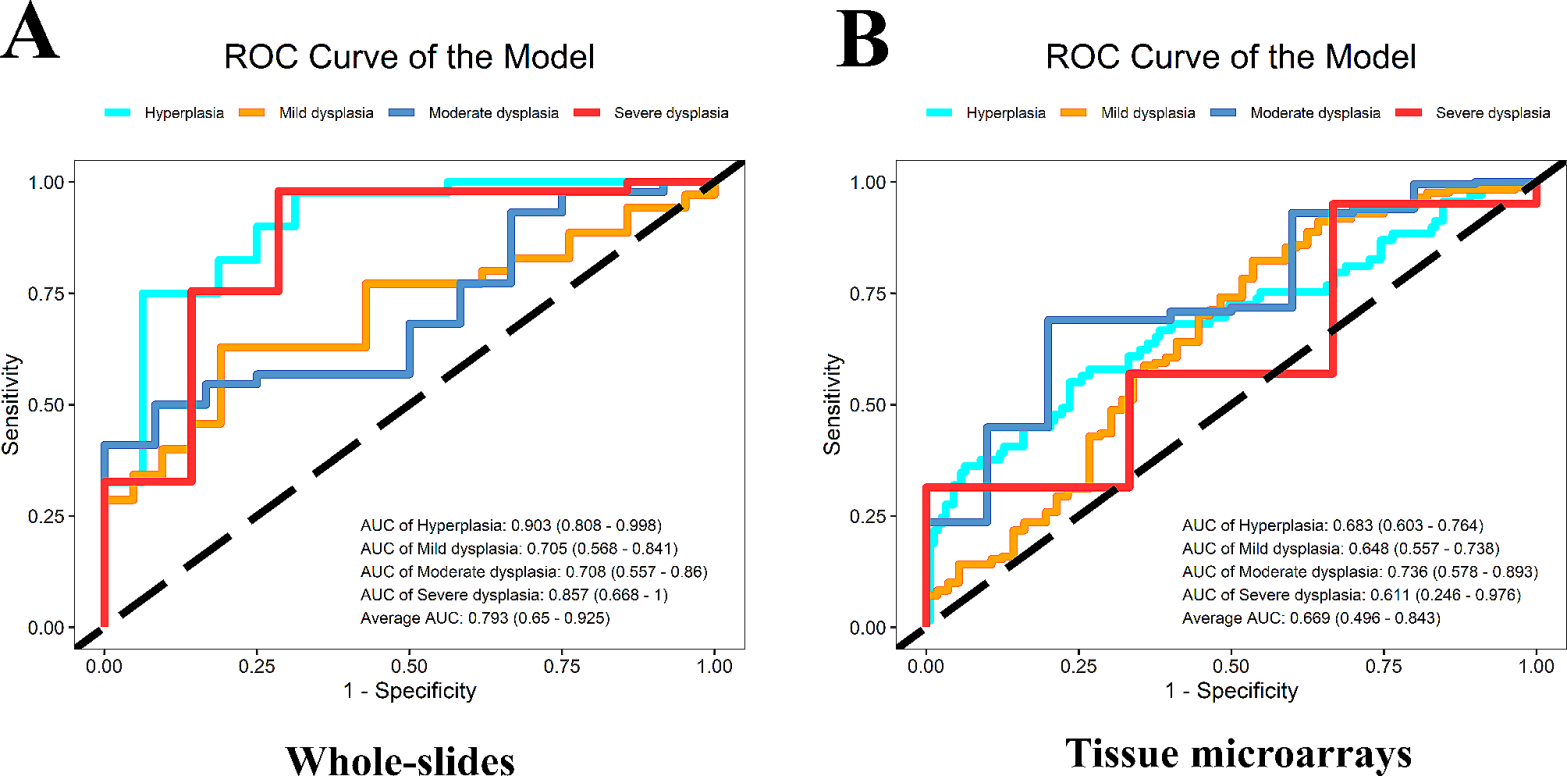
(**A**) The ROC curves of the multiclass logistic model in whole-slides. (**B**) The ROC curves of the multiclass logistic model in tissue microarrays. ROC: receiver operating characteristic; AUC: area under the curve

In the internal test dataset, the multiclass logistic model had an average accuracy of 81.3% (95% CI: 71.4%∼90.5%), an average AUC of 0.793 (95% CI: 0.650 ∼ 0.925, Fig. 3A), an average sensitivity of 61.6% (95% CI: 33.3%∼85.4%), and an average specificity of 86.0% (95% CI: 76.2%∼94.7%). We also used TMA images to test the robustness of the model. It had an average accuracy of 86.5% (95% CI: 82.4%∼90.0%), an average AUC of 0.669 (95% CI: 0.496 ∼ 0.843, Fig. 3B), an average sensitivity of 70.6% (95% CI: 67.3%∼73.9%), and an average specificity of 79.4% (95% CI: 76.1%∼82.8%). The average accuracy of the model (86.5%) was significantly higher than E-MOD (63.5%).Furthermore, this model demonstrated superior performance over three junior oral pathologists in terms of OED grading. In the internal test dataset, it achieved the highest accuracy and AUC. Meanwhile, in the TMA dataset, it exhibited the highest values for almost all metrics, including accuracy, AUC, and sensitivity (Supplementary Table 2).

**Fig. 4:**
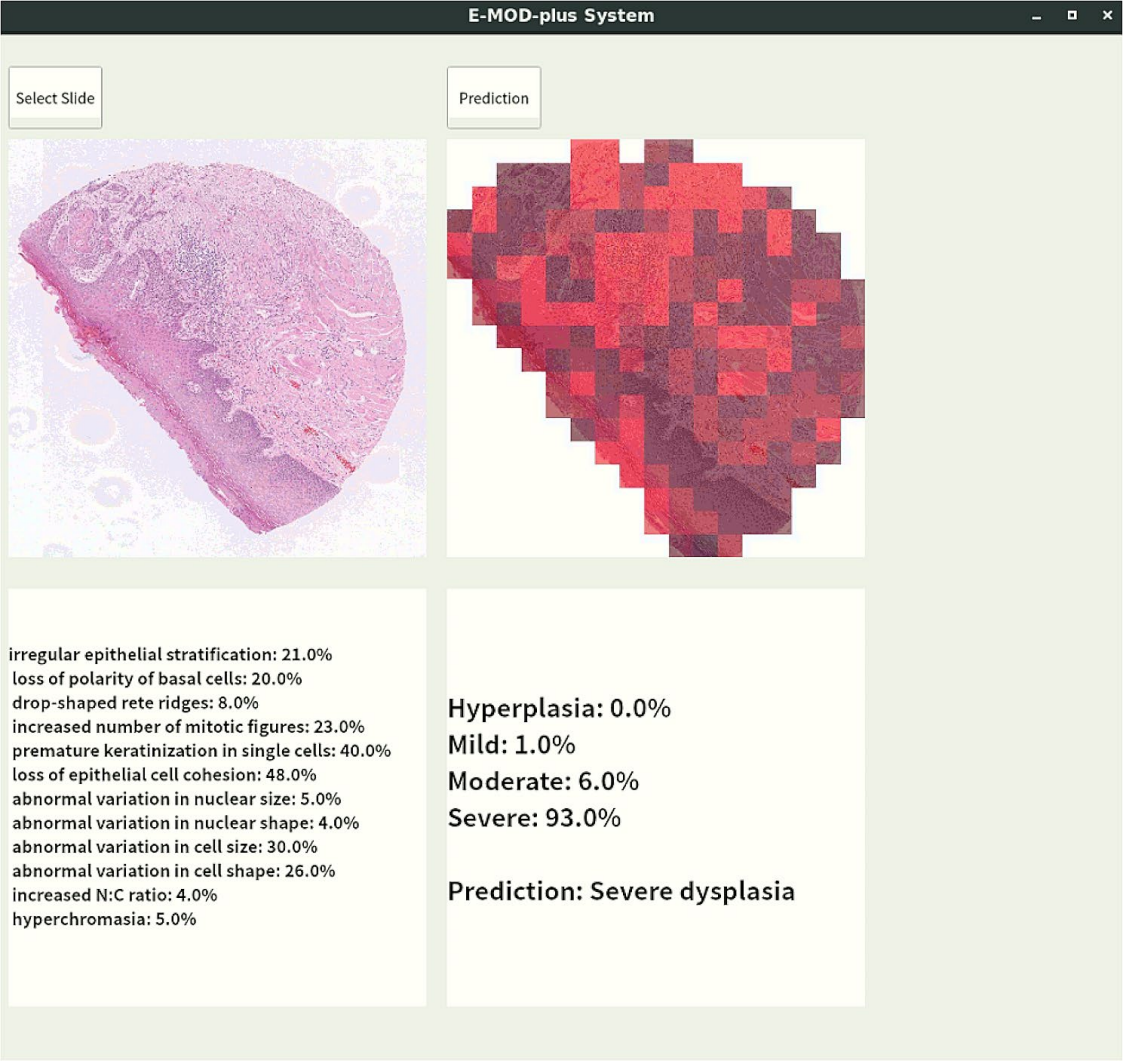
The interface of E-MOD-plus, the proposed detection and grading application of oral epithelial dysplasia. The upper left shows the input whole-slide image; the upper right shows a heatmap which marks a higher level of oral epithelial dysplasia with a brighter color; the lower left shows the probabilities of 12 pathological features; and the lower right shows a prediction of the whole-slide

Finally, we combined the model and E-MOD to establish a comprehensive grading system for OED, which was named **E-MOD-plus**. Meanwhile, an application of E-MOD-plus was developed, as an auxiliary tool for oral pathologist. As Fig. 4 shows, E-MOD-plus can not only accurately predict OED level at the whole-slide level, but also predict the presence of OED features.

## Discussion

OED grading is crucial to the prognosis of OLK, and could also influence prevention or treatment strategies [4]. While the complexity of the OED grading criteria makes the job heavy to oral pathologists, an accurate and objective computational model could be helpful for improving the efficiency of OED grading.

In our study, four CNNs were firstly evaluated using four differently scaled image inputs. All of them reached promising performances, especially EfficientNet-B0. When trained with 20×, 224-pixel patches, EfficientNet-B0 model (E-MOD) achieved the highest accuracy and AUC, at the patch-level. The selection of CNN is an important factor to develop a computer-aided grading system. ResNet-50 and Inception-V4 are two popular networks for medical image analysis due to high accuracy and robustness, but the training process is usually slow because of the complexity of their structures [29, 30]. ShuffleNet-V2 is a significantly faster network designed for mobile devices, but the accuracy is relatively lower [31]. EfficientNet-B0 is an advanced one that combines high accuracy and high speed. The essence of EfficientNet-B0 is to systematically scale depth, width and resolution, to achieve a balance of accuracy and efficiency [32]. Therefore, EfficientNet-B0 was selected as the best model for OED grading.

However, in the TMA dataset, E-MOD had an average accuracy of 63.5%, and an average AUC of 0.673, at the slide level. These results suggest that using the proportions of patches to directly integrate the classification of a deep learning model was probably not a proper approach. Hence, we considered combining the detection of OED features to assist slide-level prediction, since this process is crucial to OED grading in the routine diagnosing workflow. Now that EfficientNet-B0 was highly accurate at the patch level (including TMA patches), we utilized EfficientNet-B0 to train feature detection models, and extracted the values of activation functions as scores. The scores were finally integrated in a multiclass logistic model and it achieved better performance than E-MOD. The average accuracy of the multiclass logistic model (86.5%) was significantly higher than E-MOD (63.5%), though there is no significant differences between the AUC. These results suggest that the combination of OED features is more effective than mere whole-slides. In comparison with junior oral pathologists, this model exhibited decent performances. Notably, this model achieved the highest accuracy and AUC in both test datasets. This not only signifies its consistently high-level performance with diverse slides, but it also reinforces the potential for computational pathology applications in OED grading.

Finally, E-MOD-plus, an OED grading application was developed, by combining E-MOD and the multiclass logistic model based on the scores of OED features. E-MOD-plus was accurate, objective and user-friendly. It is noteworthy that the highlighted patches, which are indicative of high diagnostic value in the prediction map, have the potential to play a decisive role in OED grading. These regions have an increased possibility of containing OED pathological features, merit thorough review by pathologists if they cannot grade OED quickly. E-MOD-plus could be positioned as an adjunct to pathologists, especially in the initial screening and re-evaluating stages. During the initial screening stage, E-MOD-plus can grade batches of slides with ease, accelerating the OED grading process. When grading complex cases, pathologists are encouraged to review the heatmaps generated by the model, to locate regions where significant pathological features of OED may be present. This collaborative approach leverages the strengths of both human expertise and machine efficiency, creating a synergy that enhances the overall OED grading process.

This study was performed as a preliminary investigation of OLK computational histopathology which had several limitations. First of all, the reference grading criteria of OED needs to be updated. As substantial modifications were made to the pathological features in the WHO criteria (2022 version) [5], it is also necessary to evaluate the effect of added features if they are included in the model. Another limitation of our study was the relatively small sample size employed in the development and evaluation of E-MOD-plus. While the current dataset has provided valuable insights and a foundation for E-MOD-plus, its size constrains the generalizability of our findings. To address this limitation in future research, collaborative efforts with other research institutions and healthcare providers will be pursued to aggregate larger datasets, enabling a more comprehensive analysis and further validation of the model’s performance. The next limitation was that we used parent labels to categorize patches, which can make the ground truth labels noisy. However, this is an obstacle in the advances of computational pathology [36]. An ideal solution to this problem is massive manual annotations of several expert oral pathologists, which is extremely time-consuming. In this study, the neural networks still showed powerful detection and grading performances, suggesting deep learning algorithms could partially overcome the disadvantage of imbalanced and noisy ground truth labels.

Moving forward, our focus lies in gathering comprehensive prognosis information on the subjects involved in the study. This approach will enable us to track the progression of malignant transformation over time, providing better understanding of the relation between OED severity and malignant transformation. Moreover, we will utilize prognosis as an outcome for enhancing our model. By incorporating detailed prognostic data, our model can evolve beyond mere OED feature detection and OED grading, aiming to contribute meaningfully to the prediction of malignant transformation.

Last but not least, although our models reached satisfactory performances when trained from scratch, they are likely to be improved with transfer learning [37]. Transfer learning replaces randomized initial weights with ones from pretrained models using known datasets such as ImageNet [38]. It is believed that an increase in accuracy and a reduction in convergence time can be observed after applying transfer learning in model training [37, 39]. Therefore, we aimed to include transfer learning in grid search strategy in future studies.

## Conclusions

In conclusion, E-MOD-plus, the OED detection and grading system for OLK, was objective and accurate in the detection of OED pathological features as well as the grading of OED, and had potential to assist oral pathologists in clinical practice.

### Electronic supplementary material

Below is the link to the electronic supplementary material.

Supplementary Material 1

Supplementary Material 2

## Data Availability

The whole-slides and tissue microarrays are not publicly available in order to protect privacy of the patients, but are available from the corresponding author upon reasonable request. The source codes of E-MOD-plus are deposited at https://github.com/chxhstatis/E-MOD-plus.

## Abbreviations

OLK: oral leukoplakia
OPMD: oral potentially malignant disorder
OED: oral epithelial dysplasia
WHO: World Health Organization
CNN: convolutional neural network
WSI: whole-slide image
TMA: tissue microarray
AUC: area under the curve
CI: confidence interval
