# Supplementary material for: Oral epithelial dysplasia detection and grading in oral leukoplakia using deep learning
Source: BMC Oral Health. 2024 Apr 9;24:434. doi: 10.1186/s12903-024-04191-z (PMC11005210; doi:10.1186/s12903-024-04191-z)
Supplement: Supplementary file 1 — Supplementary Material 1 [file 12903_2024_4191_MOESM1_ESM.docx]

**Supplementary Table 1. An approximate number of image patches used in this study.**

| **Image Source** | **224 px & 10×** | **224 px & 20×** | **512 px & 10×** | **512 px & 20×** |
| --- | --- | --- | --- | --- |
| **WSI** | 27,000 | 109,000 | 5,000 | 20,000 |
| **TMA** | 8,000 | 31,000 | 2,000 | 6,000 |

**WSI: whole-slide image; TMA: tissue microarray; px: pixel.**
