# Supplementary material for: Oral epithelial dysplasia detection and grading in oral leukoplakia using deep learning
Source: BMC Oral Health. 2024 Apr 9;24:434. doi: 10.1186/s12903-024-04191-z (PMC11005210; doi:10.1186/s12903-024-04191-z)
Supplement: Supplementary file 2 — Supplementary Material 2 [file 12903_2024_4191_MOESM2_ESM.docx]

**Supplementary Table 2. Comparison of OED grading performance with E-MOD-plus and three junior oral pathologists.**

| **Group** | **Examiner** | **Accuracy (%)** | **95% CI** | **AUC** | **95% CI** | **Sensitivity (%)** | **95% CI** | **Specificity (%)** | **95% CI** |
| --- | --- | --- | --- | --- | --- | --- | --- | --- | --- |
| WSI | E-MOD-plus | 81.3 | 71.4~90.5 | 0.793 | 0.650~0.925 | 61.6 | 33.3~85.4 | 86.0 | 76.2~94.7 |
|  | Pathologist 1 | 54.8 | 44.5~63.8 | 0.594 | 0.462~0.709 | 40.0 | 26.5~58.5 | 69.6 | 57.1~90.2 |
|  | Pathologist 2 | 70.1 | 57.5~86.4 | 0.569 | 0.487~0.672 | 60.1 | 35.0~82.4 | 80.0 | 73.1~91.8 |
|  | Pathologist 3 | 77.7 | 68.5~89.4 | 0.656 | 0.502~0.788 | 68.8 | 39.5~90.2 | 86.6 | 75.5~96.8 |
| TMA | E-MOD-plus | 86.5 | 82.4~90.0 | 0.669 | 0.496~0.843 | 70.6 | 67.3~73.9 | 79.4 | 76.1~82.8 |
|  | Pathologist 1 | 65.1 | 50.3~78.0 | 0.532 | 0.422~0.689 | 50.0 | 39.4~60.6 | 80.2 | 75.3~84.5 |
|  | Pathologist 2 | 59.3 | 46.8~74.9 | 0.648 | 0.496~0.790 | 41.9 | 27.1~59.7 | 76.8 | 71.4~82.5 |
|  | Pathologist 3 | 70.3 | 52.5~86.9 | 0.621 | 0.487~0.775 | 67.2 | 41.0~87.4 | 73.4 | 69.5~77.7 |

**All metrics were shown as macro-average values. OED: oral epithelial dysplasia; WSI: whole-slide image; TMA: tissue microarray; CI: confidence interval; AUC: area under the receiver operating characteristic curve**
